# Supplementary material for: The gut microbiota modulates host amino acid and glutathione metabolism in mice
Source: Mol Syst Biol. 2015 Oct 16;11(10):834. doi: 10.15252/msb.20156487 (PMC4631205; doi:10.15252/msb.20156487)
Supplement: Supplementary file 1 — Expanded View Figures PDF [file msb0011-0834-sd1.pdf]

Expanded View Figures

CONV-R
GF

Tissue	CONV-R	GF
Duodenum	5	2
Jejunum	4	4
Ileum	5	4
Colon	5	4
Liver	5	3
Subcutaneous fat	4	4
Epididymal fat	5	4
TOTAL	**33**	**25**

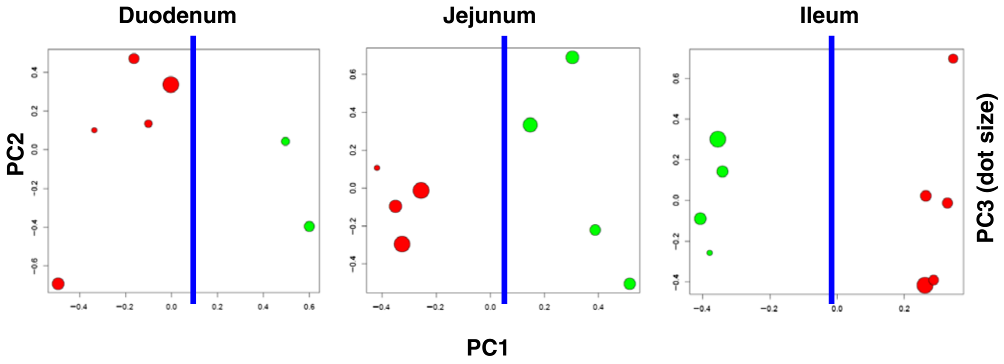

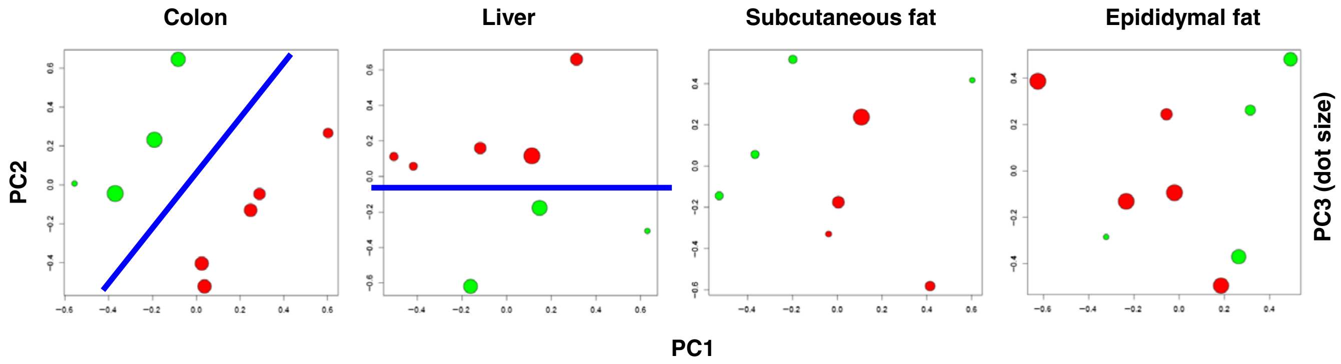

Figure EV1. Gene expression profiling of CONV-R and GF mice tissues.
Global gene expression profile of seven different tissues including liver, epididymal and subcutaneous fat, duodenum, jejunum, ileum, and colon tissues has been generated, and each tissue sample is normalized independently. Principal component analysis (PCA) of transcription profiles on each tissue is presented.

 © 2015 The Authors
